# Leveraging time for better impulse control: Longer intervals help ADHD children inhibit impulsive responses

**DOI:** 10.1371/journal.pone.0319621

**Published:** 2025-03-10

**Authors:** Inga Korolczuk, Boris Burle, Laurence Casini, Krzysztof Gerc, Dorota Lustyk, Magdalena Senderecka, Jennifer T. Coull

**Affiliations:** 1 Department of Psychology, Medical University of Lublin, Lublin, Poland; 2 Centre for Research in Psychology and Neuroscience (UMR7077), Aix-Marseille University & CNRS, Marseille, France; 3 Institute of Applied Psychology, Jagiellonian University, Kraków, Poland; 4 Centre for Cognitive Science, Jagiellonian University, Kraków, Poland; University of Hamburg, GERMANY

## Abstract

Children diagnosed with an Attention Deficit Hyperactivity Disorder (ADHD) often exhibit impulsivity and timing difficulties. Here, we investigated whether children (mean age =  9.9 years) with combined type ADHD, comprising both hyperactive-impulsive and inattentive symptoms, could use the temporal predictability of an event to help inhibit impulsive behaviour. In an adapted Simon task, we measured the effects of temporal predictability on the speed and accuracy of choice reaction times (RT) to targets appearing after short or long intervals. Temporally predictive information was conveyed either explicitly (visual cues) or implicitly (cue-target interval). Analysis of RT distributions allowed us to decompose impulsive behaviour into two key elements: the initial urge to react impulsively, and the subsequent ability to inhibit any impulsive erroneous behaviour. Both healthy controls and ADHD children could use temporal predictability conveyed by temporal cues and the length of the trial to speed their RT. However, in healthy children both explicit and implicit temporal predictability impaired inhibition of impulsive responses. In turn, although children with ADHD had stronger tendency for impulsive responding and abnormal patterns of inhibition as compared to controls, the temporal predictability of the target did not exacerbate these effects. Indeed, responding to targets appearing after long, rather than short, intervals improved inhibition in ADHD children. Taken together, our results suggest that children with ADHD can make use of longer preparatory intervals to help inhibit impulsive behaviour.

## 1. Introduction

Attention deficit hyperactivity disorder (ADHD) is a neurodevelopmental disorder that commonly manifests in childhood and can persist into adulthood. One of the hallmark challenges faced by children with ADHD is a difficulty with cognitive control, which affects their ability to regulate action effectively, often resulting in impulsive behaviour [1]. However, somewhat overshadowed by the focus on cognitive control, timing difficulties might play an equally crucial role in daily functioning of children with ADHD but are less often recognized [2]. Indeed, individuals with ADHD often struggle with various aspects of motor timing, such as production and reproduction of temporal intervals [2–16]. Timing deficits in ADHD are also found even when tasks require non-motor, perceptual estimation and discrimination of durations or intervals [17–26]. Motor and perceptual timing impairments can hinder various aspects of daily life and impact academic performance as it becomes challenging to estimate task duration or keep up with the pace of lessons.

In addition to difficulties related to motor and perceptual timing, individuals with ADHD often experience impairments in anticipatory timing, which involves the ability to predict the timing of future events or actions, enabling them to prepare and respond proactively. It can, for example, affect social interactions through interruptions and impulsive responses without due consideration of their consequences. By implicitly processing the temporal information inherent in the regular timing of the trial structure or the unfolding dynamics of time itself, it is possible to anticipate or predict when an event will occur. In neurotypical participants, RTs are faster following regular versus irregular intervals (known as the “fixed foreperiod (FP)” effect), or after long versus short intervals (the “variable FP” effect) [27–31]. In both cases, performance improves as the temporal predictability of target appearance increases. Yet relatively few studies have investigated the effects of temporal predictability on performance in ADHD children. Some studies have suggested that children and adolescents with ADHD show a reduced RT benefit from temporal predictability [32,33] while others indicate that temporal regularities do not significantly impact response speed but instead increase response variability and number of impulsive errors [5,14,24]. Indeed, temporal regularities also increase impulsive errors in adults with ADHD [34], suggesting that impairments in inhibitory control elicited by temporal regularities persist into adulthood. Moreover, the inhibition of microsaccades that usually precedes a temporally predictable target is also reduced in ADHD [35], indicating that impairments of motor inhibition by temporal predictability not only affect manual responses but also oculomotor ones. However, some recent studies present a different perspective. Thibeault et al. (2016) [36] observed that children with comorbid ADHD and Tourette’s syndrome could effectively use temporal predictability to speed their responses to regularly presented events, while keeping their impulses under control. Similarly, Vallesi et al. (2016) [37] found that children with ADHD displayed the typical pattern of faster RTs for targets appearing after long intervals compared to short ones, implying that ADHD children can implicitly form temporal predictions based on the trial’s duration to guide goal-directed actions.

The temporal predictability of an event can also be conveyed more explicitly through symbolic cues learned to be associated with specific temporal intervals. For instance, a cue in the form of a rabbit could indicate that the target will occur after a short interval, whereas a cue in the form of a tortoise would indicate that it will occur after a longer interval [38]. Typically developing children [39,40] and even infants as young as 12-15 months old [41] can effectively use temporally informative cues to speed responses to a target, a phenomenon known as temporal orienting of attention [42,43]. However, if targets are spatially unpredictable [44] or if temporal expectations vary from one trial to the next [38], then children aged 8-12 years fail to make use of symbolic temporal cues to improve response speed.

Moreover, if the temporally predictable target induces a response conflict (e.g., a target requiring a left-hand response appears on the right-hand side of the screen), the benefit of temporal cues on response speed comes at the cost of poorer response selection. In other words, temporal cues might make you so fast that you begin to make impulsive errors (e.g., automatically making a right-hand button press to a right-lateralised target even though task instructions specified a left-hand response for that particular target type). This trade-off between response speed and response selection has been observed in both healthy adults and children [45–50]. Surprisingly, however, there is a notable absence of studies investigating whether children with ADHD are able to use explicit temporal cues to guide response selection in situations of response conflict, or even whether they could use them to simply improve response speed. Thus, the goal of the current study was to examine whether children with ADHD—all diagnosed with the combined presentation, encompassing both hyperactive-impulsive and inattentive symptoms—can form and utilize temporally predictive information, conveyed either explicitly through visual cues (temporal cue effect) or implicitly by the length of the cue-target interval (variable FP effect), to effectively guide response speed and selection.

Given the known difficulties of ADHD children in overcoming impulsive tendencies, and recent evidence that temporal predictability can worsen impulse control in healthy adults and children, we investigated whether temporal predictability would further exacerbate impulsive responding in children with ADHD. To do so, we used a modified version of the Simon task [51], which required selective suppression of incorrect impulsive responses in favour of more goal-directed ones to targets that appeared either at predictable or unpredictable moments in time. Specifically, in the Simon task, participants discriminate lateralized target shapes using left or right-hand responses (e.g., pressing the left key for ‘x’ and the right key for ‘+’.) The hand assigned to a particular shape may or may not align with the target’s position on the screen, constituting “compatible” and “incompatible” conditions, respectively. During incompatible trials, participants face the challenge of suppressing the automatic tendency to respond with the hand situated on the same side as the target and must instead make a goal-directed response with the hand that was assigned to that specific shape. This response conflict results in slower RT and an increased rate of errors in incompatible trials, typically referred to as the “interference” effect, which is often used as an index of impulsive tendencies. However, the interference effect represents the outcome of dynamic interactions amongst several underlying cognitive control processes. These processes can be disentangled by employing distributional analyses of RT and accuracy, a methodology previously employed in studies of adults [52–58] and children [59–66]. Using these chronometric measures, we decomposed impulsive behaviour into two temporally and functionally distinct components: the initial automatic impulse to react quickly, but potentially incorrectly, to salient stimulus characteristics (e.g., target position), and the subsequent ability to inhibit such erroneous impulses in favour of slower, controlled responses based on task instructions (e.g., target shape). The early, automatic component of impulsivity can be revealed by Conditional Accuracy Functions (CAF), which depict accuracy as a function of RT. The CAF typically shows a decrease in accuracy for fast responses, revealing that quick reactions to incompatible targets result in more errors. This indicates that fast responses are often driven by automatic, pre-established stimulus-action associations rather than deliberate, goal-oriented processes [54]. By contrast, controlled inhibitory processing takes time to develop and so is more evident during the slowest responses [67]. Hence, as RT gets longer, the interfering effect of incompatible targets diminishes. This phenomenon is visually represented through “delta plots”, which show the size of the interference effect as a function of RT. A larger difference in the magnitude of the interference effect between fast and slow RTs (i.e., more negative-going slope) is considered to indicate more successful inhibition of impulsive tendencies.

The goal of the present study was to investigate the effects of implicit and explicit temporal predictability on action control in children diagnosed with ADHD compared to their typically developing peers. Implicit temporal predictability, based on the length of the interval (variable FP effect), is thought to require minimal attentional effort, as it is driven by automatic processes [68–70]. Since these processes are not typically impaired in ADHD, we hypothesized that children with ADHD would utilize the increased temporal predictability afforded by long intervals to guide their responses in a manner similar to that of neurotypical children [37]. Consequently, both groups of children should show faster overall RTs in long compared to short FP conditions. In contrast, since the temporal information conveyed explicitly by visual cues requires more voluntary attention, we hypothesised that children with ADHD may not use these cues as effectively as typically developing children, resulting in a significant reduction in the RT benefit of the temporal (versus neutral) cue in ADHD versus typically developing children.

Nevertheless, when the response to a temporally predictable target is incompatible with its position on the screen, the RT benefits of temporal predictability are accompanied by a higher number of fast impulsive errors, and poorer inhibition of these impulses in neurotypical adults and children [47–50]. We therefore expected that in typically developing children both implicit and explicit temporal predictability would increase the number of impulsive errors as shown by lower accuracy for the fastest responses to incompatible targets (CAF analysis). In parallel, temporal predictability would also lead to greater difficulty in inhibiting these impulsive errors, manifested by a large interference effect even when responses were very slow (less negative-going delta plots). By contrast, given that children with ADHD make more premature responses to temporally predictable targets [71] and have greater difficulty suppressing impulsive actions to incompatible targets in general [62,63], we hypothesised that the effects of both implicit and explicit temporal predictability on responses to incompatible targets would be exacerbated in children with ADHD. More specifically, we expected that long FPs, as well as temporal cues, would further increase the number of fast impulsive errors measured by the CAF and further impair inhibition of these impulses, indexed by a larger interference effect in the last segment of the delta plot.

## 2. Methods

### 2.1. Participants

We tested 40 children. The ADHD group consisted of 20 participants (mean age =  9.85 years, *SD* =  1.35 years, range =  8-13 years, 19 males). The control group consisted of 20 typically developing children (mean age =  9.9 years, *SD* =  1.37 years, range =  8-13 years, 19 males). Children were matched for age and sex. The maximal age difference between a child with ADHD and a demographically matched control was 4 months. Sample sizes were based on prior studies examining the effects of temporal predictability on performance [39,72] and inhibitory control [59,73] in typically developing children as well as in children diagnosed with ADHD [36,37,62,63].

ADHD children were referred to the study by the local diagnostic and counselling centre for child development (Skawina, Poland). All of them were diagnosed with a combined hyperactive-impulsive and inattentive type of ADHD based on the DSM-V criteria (i.e., six or more symptoms of hyperactivity/impulsivity and six or more symptoms of inattention). The diagnosis was conducted independently by a psychologist and a physician, in line with the current evidence-based diagnosis guidelines [74], and involved incorporating diagnoses from additional raters who know the child in other contexts (e.g., teachers and parents). Children had no history of neurological, psychiatric or medical disorders. Three children displayed symptoms of oppositional defiant disorder (ODD), a common comorbidity in ADHD. All participants were not receiving any medication at the time of testing; however, two children had previously received medication for their ADHD (one methylphenidate, and another hydroxyzine and risperidone). Participants had an IQ of 80 or higher on the Stanford–Binet Intelligence Scales – 5th Edition [75].

Control participants were recruited from local schools (Katowice, Poland). They had no diagnosis of ADHD or related disorders, as reported by parents and teachers. While they did not undergo the full diagnostic procedure used for the ADHD group, we implemented a careful screening process: parents were asked if their child had ever been evaluated or treated for attentional or hyperactivity issues, and only those with no reported concerns or prior indications of ADHD were included.. All participants had normal or corrected-to-normal vision. Children gave verbal assent before the experiment, which was witnessed by both the parent and the experimenter, and noted in the study protocol. Children’s legal guardians provided formal written consent. Ethics approval was obtained from the University Research Ethics Committee of the Institute of Philosophy at Jagiellonian University in Krakow, Poland, on 25^th^ June 2020. The recruitment period for this study was 20^th^ August 2020 to 29^th^ April 2021.

### 2.2. Experimental task

**Fig 1:**
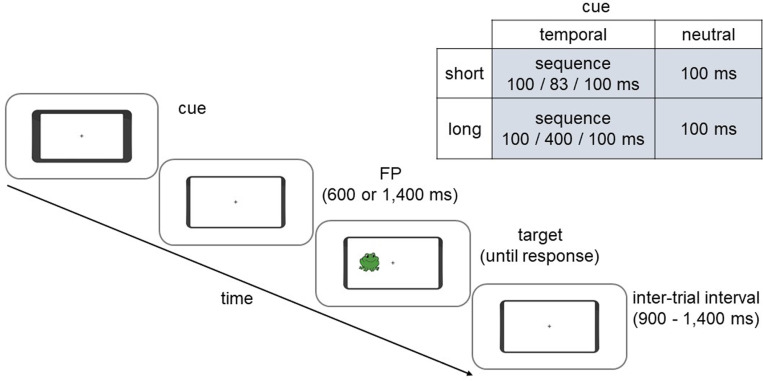
The trial timeline of the temporally cued Simon task. A cue provided information (temporal condition) or not (neutral condition) about the time of target occurrence. The background display consisted of the outline of a mobile phone and a central fixation cross, and were present throughout the task. In the temporal cue condition, the phone outline briefly (100 ms) flashed twice, with the flashes separated by either an 83 ms (short FP trials) or 400 ms (long FP trials) empty interval. In the neutral cue condition, the phone outline flashed briefly (100 ms) once. During the subsequent FP, the background display was presented for either 600 ms (short FP trials) or 1,400 ms (long FP trials). Next, a target (pig or frog) appeared on either the right or left side of the fixation cross and participants made their choice response (right or left index finger for frog or pig, counterbalanced across participants). The inter-trial interval was randomised between 900-1,400 ms.

All participants performed a temporally cued version of the Simon task with child-friendly stimuli, as used in Ambrosi et al. (2020). The task was presented using PsychoPy software [76]. The background visual display consisted of a black central fixation cross (0.5°) surrounded by a black centrally located rectangular frame (14.8° ×  6.9°) that resembled the outline of a mobile phone, presented against a white background (Fig 1). Both the phone outline and the fixation cross (0.5°) remained on the screen throughout the task. Targets were green frog and pink pig cartoon stimuli (1.7° ×  1.7°), which appeared either on the left or right side of the fixation cross at a distance of approximately 3.6° of visual angle.

There were two cue conditions presented in two separate blocks (counterbalanced across participants): temporal and neutral. Although young children [38,39], and even babies [41], can use symbolic temporal cues to predict when a target is likely to occur, children as old as 10-12 years find it hard to effectively use such cues if target locations [44] or temporal expectations [38] vary from one trial to another. Therefore, we could not use a symbolic temporal cue to assess the effects of ADHD on performance in the Simon task, since both the temporal predictability and spatial location of the target vary on a trial-by-trial basis in this paradigm. Instead, to elicit robust temporal cueing effects in our sample of 10 year olds, temporal expectations were induced by the duration of the interval between two brief flashes of the phone outline [72]. During each brief flash (100 ms each) the phone outline slightly thickened. These two flashes were separated by either a short (83 ms) or long (400 ms) empty interval or “foreperiod” (FP), during which the background display remained on the screen. In short FP trials, the duration of the empty interval (83 ms) informed participants that a target would occur soon (after 600 ms). In long FP trials, the duration of the empty interval (400 ms) indicated that a target would occur later (after 1,400 ms). Importantly, to minimise rhythmic entrainment effects, the duration of the empty interval was not a multiple of the subsequent FP. Children could use these temporal cues (T) to form predictions about the time of target onset. All temporal cues were valid. By contrast, the neutral cue (N) consisted of a brief (100 ms) thickening of the phone outline, followed by either a 600 ms or 1400 ms FP. The neutral cue therefore provided no temporally precise information about target onset.

Children were asked to use the information provided by the cue (“flickering of the mobile phone’) to respond as quickly and accurately as possible according to the shape (frog or pig) of the target (“take a picture of the animal using the correct button”) [39]. Children were instructed that if it was a short flicker then the target would appear soon, or if it was a long flicker the target would appear later. Half of the participants pressed ‘lctrl’ on a standard QWERTY keyboard with their left index finger for the “frog” and ‘>’ with their right index finger for the “pig”. The finger-response pairings were reversed for the remaining participants. A target could be presented on either the right or left side of the fixation cross. Thus, the side of the target presentation could be either the same as (compatible condition) or opposite to (incompatible condition) the response hand.

The trial’s structure was as follows. The trial started with presentation of the cue (T or N). During the temporal condition, the cue sequence was presented for 283 ms in total for the short FP trials and 600 ms in total for the long FP trials. During the neutral condition, the cue was presented for 100 ms. After the cue, the background display was presented for one of the two FPs (600 or 1,400 ms). Then, the target appeared and remained on the screen until a response was given. During the inter-trial interval, which varied between 900 – 1,400 ms (in steps of 100 ms), the background display was presented.

Each of two blocks (T and N) contained 120 trials. In each block, the proportion of short (600 ms) and long (1,400 ms) FPs was 50:50, and the proportion of compatible to incompatible trials was 50:50, presented in a randomised order. Altogether, there were 30 trials per each of the 8 combinations of the cue, FP and compatibility conditions. Breaks were given every 20 trials. A training session was provided during which participants performed 72 trials to familiarise them with the task. In the first part of the training session, participants completed a series of 6 temporal short trials, followed by a series of 6 temporal long trials, then a series of 12 mixed temporal short and long trials, and finally a series of 12 neutral trials. Auditory feedback was given after each response. In the second part of the training session participants did not receive auditory feedback while they completed 24 mixed temporal trials followed by a series of 12 neutral trials. Although we did not set a strict performance-based criterion for transitioning from practice to the main task, the structured training session was designed to ensure participants understood the task requirements. Participants first received immediate feedback during a graded introduction—progressing from simple to more complex trial sequences—before performing a second training phase without feedback. This approach allowed us to confirm that participants understood the instructions and could execute the required responses consistently. If any participant appeared unsure or had persistent difficulties during training, the experimenter explained the instructions and procedure again before moving on to the main task.

### 2.3. Data analysis

Since our experiment employed only two foreperiods (FP) and no catch trials, the absence of a target at the short FP in the neutral condition meant that participants could predict with 100% certainty that the target was therefore bound to occur at the long FP [77]. This rendered temporal predictability similar in both temporal and neutral conditions for long FP trials. Consequently, to assess the impact of temporal cues, we focused exclusively on the data from short FP trials only. To measure the effects of the variable FP effect, we analysed data from neutral cue trials only.

Given that children are prone to lapses in attention, which can lead both to overly delayed responses and to anticipatory responses, we trimmed the dataset by removing response times (RTs) that fell below or exceeded 3 standard deviations from each participant’s mean (Table 1). In order to measure the effect of temporal cues on correct RT, a three-way mixed ANOVA, with group (ADHD/control) as a between-subjects factor, and cue (temporal/neutral) and compatibility (compatible/incompatible) as within-subjects factors, was conducted. The percentage of errors was submitted to a three-way mixed ANOVA comprising group (ADHD/control) as a between-subjects factor and cue (temporal/neutral) and compatibility (compatible/incompatible) as within-subjects factors. To assess the effect of the variable FP effect on correct RT, we performed a three-way mixed ANOVA, with group (ADHD/control) as a between-subjects factor, and FP (short/long) and compatibility (compatible/incompatible) as within-subjects factors. The effect of the variable FP effect on the percentage of errors was assessed by means of a three-way mixed ANOVA comprising group (ADHD/control) as a between-subjects factor and FP (short/long) and compatibility (compatible/incompatible) as within-subjects factors.

**Table 1:** Percentages of trials excluded from the analyses due to anticipatory or excessive RT.

Group	% Anticipatory RT (mean in ms)	% Excessive RT (mean in ms)
Matched Controls	0.15% (178)	1.46% (1979)
ADHD	0.17% (155)	1.58% (2882)

To reveal the dynamics of committing errors we computed Conditional Accuracy Functions (CAF), which plot accuracy as a function of RT. This analysis is based on a vincentisation of the data [78,79]. For each participant and for each of the four temporal conditions (temporal/neutral cue ×  short/long FP) and two compatibility conditions (compatible/incompatible target), RTs from both correct and incorrect trials were ranked in ascending order and grouped into five bins of equal size (quintiles). The percentage of correct responses in each quantile was quantified and used as a dependent variable. In order to reveal the effects of temporal cues on the probability of correct response as a function of RT, these percentages were submitted to a four-way mixed ANOVA involving group (ADHD/control) as a between-subjects factor and cue (temporal/neutral), compatibility (compatible/incompatible) and quintile (1 to 5) as within-subjects factors. The effect of the variable FP effect on the probability of correct response as a function of RT was assessed using a four-way mixed ANOVA involving group (ADHD/control) as a between-subjects factor and FP (short/long), compatibility (compatible/incompatible) and quintile (1 to 5) as within-subjects factors.

To measure the effects of temporal predictability on the dynamics of the interference effect (incompatible – compatible RT) we calculated delta plots, which depict the interference effect as a function of RT [67,80,81]. Delta plots are also conducted using vincentised RTs but from correct trials only. Correct RTs were ranked in ascending order grouped into five quintiles containing the same number of trials. Next, the difference in mean RT between incompatible and compatible trials (delta value) was extracted for each quantile, separately for each participant, and for each of the four temporal conditions. To reveal the effects of temporal cues on the delta plots, these delta values were entered into a three-way mixed ANOVA involving group (ADHD/control) as a between-subjects factor and cue (temporal/neutral), and quintile (1 to 5) as within-subjects factors. The effects of the variable FP effect were measured by the means of a three-way mixed ANOVA involving group (ADHD/control) as a between-subjects factor and FP (short/long), and quintile (1 to 5) as within-subjects factors. These analyses were then supplemented with analysis of the final segment of the delta plot (i.e., between quintile 4 and quintile 5), which is thought to index the slow voluntary inhibition of the fast automatic impulse. Specifically, the slopes of the final segment of the delta plot were entered into a two-way mixed ANOVA involving group (ADHD/control) as a between-subjects factor and cue (temporal/neutral) as a within-subjects factor to reveal the effects of temporal cues, and a two-way mixed ANOVA involving group (ADHD/control) as a between-subjects factor and FP (short/long) as a within-subjects factor to reveal the effects of the variable FP effect.

## 3. Results

### 3.1. Response times

#### 3.1.1. Temporal cueing.

RT results are based on the correct trial data. As expected, we found a main effect of compatibility on RT, *F*(1, 38) =  35.83, *p* < .001*,* η_p_^2^ = .49. RTs were slower for incompatible versus compatible targets for both control and ADHD groups. A significant main effect of cue, *F*(1, 38) =  5.51, *p* = .024*,* η_p_^2^ = .13, was further qualified as a significant Cue ×  Compatibility interaction, *F*(1, 38) =  9.92, *p* = .003, η_p_^2^ = .21. Replicating previous results in adults [47], temporal cues speeded RTs to compatible targets (*p* = .001) but not incompatible ones (*p* =  0.351). In other words, the benefit of temporal cueing was observed only when there was no conflict about what to respond. A Group ×  Cue ×  Compatibility interaction was far from reaching statistical significance, *F*(1, 38) =  0.08, *p* = .776. There was no main effect of group, *F*(1, 38) =  1.97, *p* = .167, or a Group ×  Compatibility interaction, *F*(1, 38) =  2.19, *p* = .148.

#### 3.1.2. Variable FP effect.

**Fig 2:**
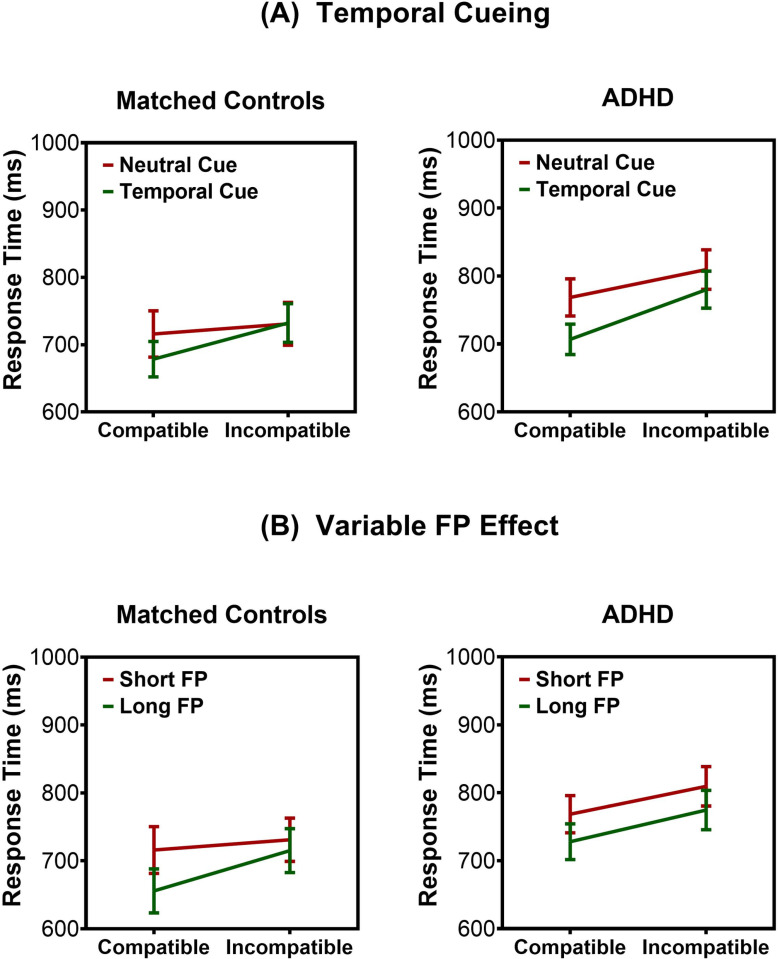
Mean response times. **(A)** Temporal cueing speeded RTs to compatible, but not incompatible, targets in both the control and ADHD groups. **(B)** In turn, the higher temporal predictability of targets appearing after the long FP led to faster RTs to compatible targets in the control group but did not interact with compatibility in the ADHD group. Error bars reflect standard errors.

The analysis of neutral trials also revealed a main effect of compatibility on RT, *F*(1, 38) =  51.74, *p* < .001*,* η_p_^2^ = .58. We observed a main effect of FP, *F*(1, 38) =  35.64, *p* < .001, η_p_^2^ = .48, and a FP ×  Compatibility interaction, *F*(1, 38) =  6.98, *p* = .012, η_p_^2^ = .16. Importantly, there was a significant Group ×  FP ×  Compatibility interaction, *F*(1, 38) =  4.15, *p* = .049, η_p_^2^ = .10. This interaction was broken down by group. In the control group, we observed a significant FP ×  Compatibility interaction, *F*(1, 19) =  16.1, *p* < .001, η_p_^2^ = .46. As expected [30,39,68,69,82–86], RTs were faster following long than short FPs, i.e., when temporal expectancy was stronger. However, similar to the effects of temporal cues reported above, this was true only for compatible targets (*p* < .001) not incompatible ones (*p* = .374) (Fig 2B). By contrast, in the ADHD group, RTs were faster after long than short FPs *F*(1, 19) =  11.22, *p* = .003, η_p_^2^ = .37, but we failed to observe a significant FP ×  Compatibility interaction, *F*(1, 19) =  0.14, *p* = .715. This shows that ADHD children processed the temporal predictability of the long FP and used it to speed responses generally, whether a target was compatible or not. No main effect of group, *F*(1, 38) =  2.45, *p* = .126, or a Group ×  Compatibility interaction, *F*(1, 38) =  0.34, *p* = .564 was observed.

### 3.2. Accuracy

#### 3.2.1. Temporal cueing.

Table 2 shows mean accuracy in the temporally cued Simon task. The analysis of error rates showed a significant main effect of compatibility, *F*(1, 38) =  25.85, *p* < .001*,* η_p_^2^ = .41, with more errors to incompatible than compatible targets. There was also a significant Group ×  Compatibility interaction, *F*(1, 38) =  9.49, *p* = .004, η_p_^2^ = .20. Children with ADHD made more errors to incompatible targets than healthy controls (*p* = .023). No other significant main effects or interactions were observed.

**Table 2:** Mean (and standard error) percentage of errors.

Group	Compatibility	FP	Temporal Cue	Neutral Cue
ADHD	Compatible	Short	3.2 (1.01)	4.5 (1.29)
Control			4.1 (0.83)	3.1 (0.78)
ADHD		Long	3.3 (1.13)	2.8 (0.85)
Control			1.3 (0.51)	3.0 (1.11)
ADHD	Incompatible	Short	8.2 (0.91)	8.0 (1.04)
Control			4.2 (1.83)	5.0 (1.6)
ADHD		Long	6.3 (1.28)	6.2 (0.97)
Control			3.3 (1.39)	4.4 (1.39)

#### 3.2.2. Variable FP effect.

Again, there was a significant main effect of compatibility, *F*(1, 38) =  17.98, *p* < .001*,* η_p_^2^ = .32. Children were less accurate in incompatible than compatible trials. No other significant main effects or interactions were noted.

### 3.3. Activation of impulsive responses (CAF)

#### 3.3.1. Temporal cueing.

**Fig 3:**
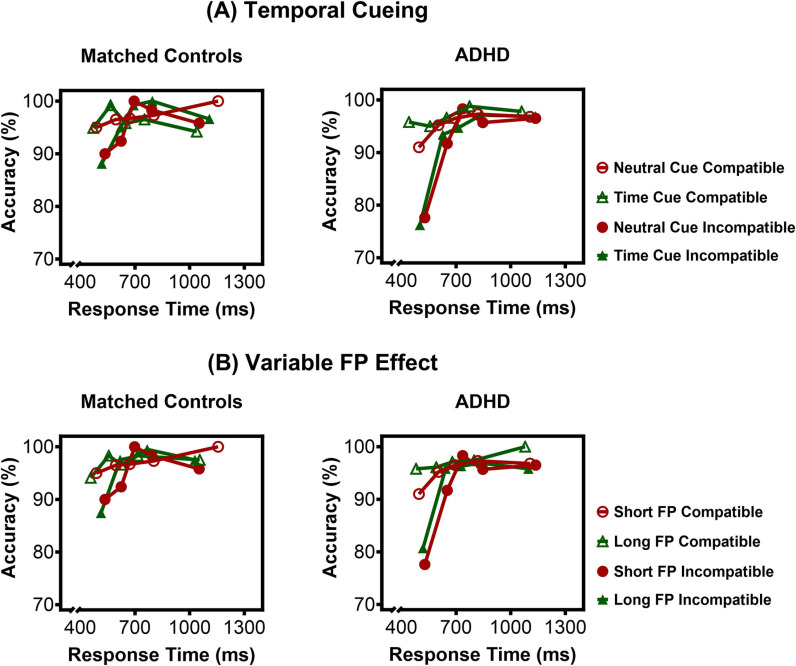
Conditional Accuracy Functions (CAF) depicting impulsive response activation as a function of RT. The plots show the probability of a correct response as a function of mean RT in each of five quintiles across participants, for both (A) cue (temporal, neutral) and compatibility (compatible, incompatible) conditions, and (B) FP (short, long) and compatibility (compatible, incompatible). ADHD children made more very fast errors overall. However, there were no further effects of temporal cueing (A) or the variable FP effect (B) on the rates of fast errors in ADHD and control groups.

To explore how the temporal predictability of cues affected the dynamics of making an error, we plotted Conditional Accuracy Functions (CAF), which show accuracy rates as a function of RT (Fig 3A). There were main effects of compatibility, *F*(1, 38) =  27.19, *p* < .001, η_p_^2^ = .42, and quintile, *F*(4, 152) =  25.75, *p* < .001, η_p_^2^ = .40. As expected, and replicating previous reports [63,67,87,88], there was a Quintile ×  Compatibility interaction, *F*(4, 152) =  12.19, *p* < .001, η_p_^2^ = .24. Accuracy was lower for incompatible than compatible targets only in quintile 1 (*p* < .001), demonstrating a transient susceptibility to respond incorrectly in conflict situations only when making very fast responses. Significant Group ×  Compatibility, *F*(1, 38) =  9.87, *p* = .003, η_p_^2^ = .21, and Group ×  Quintile, *F*(4, 152) =  3.49, *p* = .009, η_p_^2^ = .08, interactions, were further explained by a Group ×  Compatibility ×  Quintile interaction, *F*(4, 152) =  2.65, *p* = .036, η_p_^2^ = .07. ADHD children made significantly more errors to incompatible targets in quintile 1 than controls (*p* = .009), showing that they have a stronger urge to rapidly execute prepotent and inappropriate responses [62,63]. There was no Cue ×  Compatibility ×  Quintile interaction, *F*(4, 152) =  1.07, *p* = .374, η_p_^2^ = .03, nor a Group ×  Cue ×  Compatibility ×  Quintile interaction, *F*(4, 152) =  0.41, *p* = .802, η_p_^2^ = .01. The results show that temporal cues did not further increase the tendency to make fast errors in control or ADHD children.

#### 3.3.2. Variable FP effect.

There was a main effect of compatibility, *F*(1, 38) =  15.30, *p* < .001, η_p_^2^ = .29, and quintile, *F*(4, 152) =  22.60, *p* < .001, η_p_^2^ = .37, further explained by a Compatibility ×  Quintile interaction, *F*(4, 152) =  11.56, *p* < .001, η_p_^2^ = .23. Again, significantly more errors to incompatible targets were observed for the fastest reaction times (quintile 1) (*p* < .001) (Fig 3B). No FP ×  Compatibility ×  Quintile interaction, *F*(4, 152) =  0.56, *p* = .697, η_p_^2^ = .01, nor a Group ×  FP ×  Compatibility ×  Quintile interaction, *F*(4, 152) =  0.42, *p* = .796, η_p_^2^ = .01, were observed. Thus, the temporal predictability of the long interval did not further increase the tendency to act impulsively, for either healthy children or those with ADHD. All other effects were also non-significant.

### 3.4. Selective inhibition of impulsive responses (delta plots)

#### 3.4.1. Temporal cueing.

**Fig 4:**
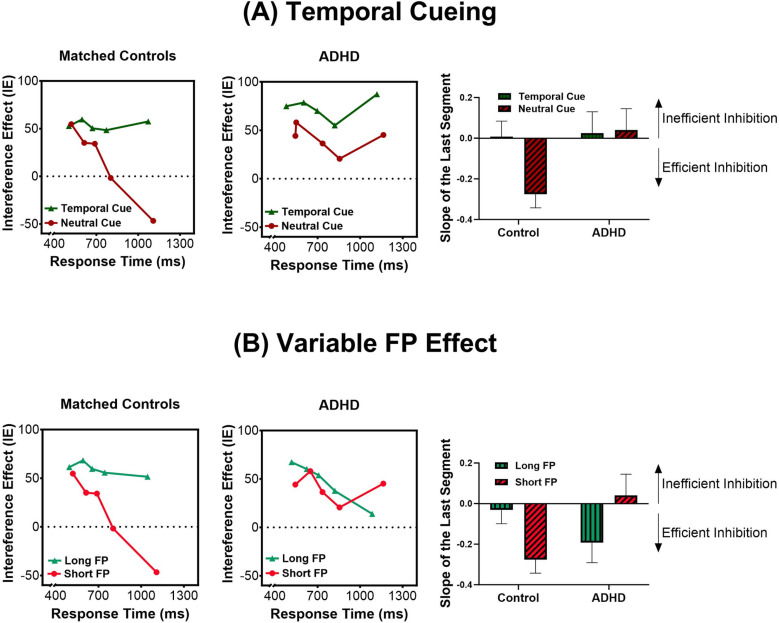
The efficiency of selective response inhibition revealed by delta plots, which depict the magnitude of the interference effect (incompatible-compatible) as a function of RT. Typically, a negative slope indicates a smaller interference effect, and thus more efficient inhibitory processing, as RTs get longer **(A)** Left: Matched controls showed the typical pattern of inhibitory control in the neutral cue condition (dark red), but had impaired inhibitory processing in the temporal cue condition (dark green). Right: By contrast, ADHD children demonstrated impaired inhibitory control in both temporal (dark green) *and* neutral (dark green) conditions. **(B)** Left: In controls, inhibitory processing deteriorated when targets appeared after long (light green) rather than short FPs (light red). Right: By contrast, inhibitory control improved for targets appearing after long FPs (light green) in the ADHD group.

We measured the effect of temporal cueing on the dynamics of the interference effect using delta plots, which depict the magnitude of the interference effect (i.e., incompatible RT-compatible RT, “delta value”) as a function of RT (Fig 4). Typically, in the Simon task, the interference effect diminishes as the response time of the participant increases. Since it takes additional processing time to inhibit a response, trials in which RTs are longest allow sufficient time for a response to be inhibited. Therefore, interference effects are smaller for the slowest responses as illustrated by a negative-going slope in the delta plots. The analysis of the mean interference effect revealed a significant Cue ×  Quintile interaction, *F*(4, 152) =  3.48, *p* = .009, η_p_^2^ = .08. The interference effect during the slowest responses (quintile 5) was greater after temporal than neutral cues (*p* = .012) (Fig 4A). A Group ×  Cue ×  Quintile interaction was close to significance, *F*(4, 152) =  2.10, *p* =  0.083, η_p_^2^ = .05.

Nevertheless, the comparison of slopes in the last segment of the delta plot did show a significant Group ×  Cue interaction, *F*(1, 38) =  4.51, *p* = .040, η_p_^2^ = .11. In the control group, we observed the expected negative-going slope following neutral cues but not temporal ones (*p* = .007) (Fig 4A). This indicates that temporal cueing impaired response inhibition in healthy children. However, the ADHD group showed an atypical upward slope in the last segment of the delta plot after both temporal and neutral cues, with no difference in slope values between the two cue conditions (*p* =  0.873). This upward slope has been observed previously in children with ADHD and reflects their difficulty in inhibiting impulsive responses [62]. Thus, ADHD children had difficulties inhibiting impulses whether or not targets were temporally predictable, while controls showed inhibition problems only when they were temporally cued.

#### 3.4.2. Variable FP effect.

The analysis of the evolution of the interference effect showed a Group ×  FP ×  Quintile interaction, *F*(4, 152) =  3.72, *p* = .006, η_p_^2^ = .09. We broke down the interaction by group. In the control group, we found a significant FP ×  Quintile interaction, *F*(4, 76) =  4.43, *p* = .003, η_p_^2^ = .189. The magnitude of the interference effect for the slowest responses (quintile 5) was greater following long versus short FP (*p* = .008) (Fig 4A). Thus, similar to the effects of temporal cues, the variable foreperiod (FP) effect led to impaired inhibition of impulsive responses in healthy children. In contrast, in the ADHD group, there was no interaction between FP and quintile, *F*(4, 76) =  0.89, *p* = .472 (Fig 4B).

The analysis of the last segment of the slope revealed a Group ×  FP interaction, *F*(1, 38) =  9.60, *p* = .004, η_p_^2^ = .20. In the control group there was a negative-going slope following short FP but not long FP (*p* = .031), (Fig 4B). Conversely, in the ADHD group, the atypical upward slope was observed in short FP but not long FP trials (*p* = .039). These results show that healthy children have difficulty inhibiting impulsive responses to targets appearing after long (temporally predictable) FPs. Conversely, ADHD children, who exhibit poor inhibition in short FP trials, appear to benefit from the longer preparatory time provided in long FP trials.

## 4. Discussion

This study investigated whether children diagnosed with ADHD exhibit dissociable patterns of action control in comparison to typically developing children when reacting to temporally predictable events. Temporally predictive information was conveyed either implicitly by the length of the cue-target interval (variable FP effect) or explicitly by symbolic cues. In contrast to previous research focusing on tasks with simple stimulus-response associations [32,33,35], our study examined the effects of temporal predictability on response speed and accuracy when the appropriate response required inhibition of a prepotent, but incorrect, action. We used sophisticated chronometric measures to decompose impulsive behaviour into the initial urge to make a fast yet incorrect response, and the subsequent ability to inhibit these impulses as measured by the strength of the interference effect in delta plots. Specifically, the interference effect, characterized by slower RTs for incompatible targets compared to compatible ones, usually diminishes as RTs become longer. This is observed as a negative slope in the delta plot, where the slope’s steepness indicates a higher level of inhibitory control. Traditional interpretation suggests that longer RTs provide participants with greater processing time with which to suppress potentially incorrect responses, leading to a reduced difference between compatible and incompatible trials [65,67,80].

When the target location corresponded with the assigned response hand (compatible condition), both implicit and explicit temporal predictability affected response speed (mean RT) similarly in both typically developing children and children with ADHD. Whether predictability was manipulated by the length of the FP or a symbolic cue, the temporal predictability of a compatible target speeded RT. These data replicate the previous work of Vallesi et al., 2016 by showing that mean RT to compatible targets was faster following long rather than short FPs in children with ADHD. We also show that RTs to compatible targets were faster following temporal, than neutral, cues demonstrating, for the first time, that children with ADHD are able to voluntarily attend to symbolic cues in order to explicitly form and use temporal predictions in the service of goal-directed behaviour.

However, group differences became apparent when we used RT distribution analyses to analyse the trade-off between response speed and response inhibition whenever the target location was *incompatible* with the assigned response hand. First, we replicated prior findings by showing that ADHD children generally exhibit poor inhibition of the incorrect response hand. We found these effects regardless of whether the target had been preceded by a temporal or a neutral cue. These atypical inhibitory patterns were exemplified by a sudden reversal in slope in the last segment of the delta plot, indicating a large interference effect even when RTs were relatively long. The pattern of effect is remarkably consistent with prior findings by Grandjean, Suarez, Miquee, et al. (2021) in a non-cued Simon task and provides further evidence that a deficit in inhibitory control might be a central factor contributing to difficulties in flexible goal-directed behaviour in individuals with ADHD [1,89,90].

Nevertheless, analysis of the last-segment slope of the delta plots also revealed that when ADHD children were provided with more time between the neutral cue and a target (long FP trials), they exhibited a more typical pattern of inhibitory behaviour (negative slope). Therefore, temporally predictable information that that was extracted implicitly during the course of the FP was more beneficial to ADHD children than provided explicitly by symbolic cues. Recent psychopharmacological research in healthy participants has also demonstrated that the effects of dopamine interventions on the performance benefits of temporal predictability are more apparent when temporal predictability has been extracted implicitly from FP duration [91] rather than explicitly via symbolic cues [92]. Given evidence from genetic and brain imaging studies linking altered dopamine signalling to various ADHD symptoms [see [93] for a review] these psychopharmacological findings converge with our own results to suggest that dopamine might be more involved in the use of posterior probabilities to guide behaviour (dynamic changes in conditional probabilities throughout the FP), rather than prior probabilities (fixed predictions provided *a priori* by the temporal cue).

By contrast to the profile of performance in ADHD children, long FPs *impaired* inhibition of incorrect responses to incompatible targets in neurotypical children. This effect was illustrated by the absence of the typical downward slope in the delta plots for long FP versus short FP targets and, in fact, exactly the same pattern was seen when comparing the effects of temporal to neutral cues in this group. These convergent findings suggest that healthy children found it more difficult to inhibit erroneous impulses whenever they could predict when the target was going to appear, whether they were processing temporal information implicitly or explicitly. Moreover, the overall pattern of results suggests that the temporal predictability of target onset makes it as difficult for neurotypical children to inhibit impulsive responses, as for children with ADHD whether the target is predictable or not. Although the behavioural effects of temporal predictability have not been observed on delta plots previously [47], temporal cueing has been shown to affect EEG indices of inhibition [49,50]. Using the same temporally-cued Simon task as in our current study, it was found that temporal predictability reduced cortical inhibition of the inappropriate, but prepotent, response to incompatible stimuli. This closely aligns with current behavioural delta plot data and indicates potential mechanisms and pathways that could contribute to impairments in inhibitory control triggered by temporal predictability.

By contrast, the CAF data demonstrated that although children with ADHD made more fast impulsive responses than healthy children, temporal predictability did not further exacerbate this effect. These findings align with recent data [36], which also showed that temporal predictability did not increase the number of premature responses in individuals with both tic disorder and ADHD. The clear distinction between the differential effects of temporal predictability on the initial tendency to act impulsively, and the subsequent ability to suppress these impulses, in ADHD suggests a selective role of dopamine in the inhibition rather than activation of impulsive responses. Indeed, this aligns with recent findings indicating that methylphenidate selectively affects response inhibition, not activation, in ADHD [62]. Similarly, individuals with Parkinson’s Disease, characterised by abnormalities in dopamine function, exhibit deficits in response inhibition without concurrent impairments in activation [53]. This further highlights the importance of chronometric measures in understanding impulsive behaviour [47,53,62,63] and complements neuroimaging research suggesting that different areas of the brain are involved in these two distinct mechanisms [94]. It should be noted, however, that previous studies [47,48] have found significant effects of temporal cues on the activation of impulsive responses. One potential explanation for the lack of effect in the current study (at least in the context of the ADHD children) could be the presence of a ceiling effect, wherein participants may have reached their maximal level of impulse capture, limiting the detectability of additional influences.

Finally, the current study presents some limitations. We tested a relatively small sample size, so future studies should include more participants. Nevertheless, our data showed patterns consistent with previous findings in children with ADHD. Specifically, children with ADHD made more errors overall. Although performance appeared close to ceiling for both groups, this pattern is consistent with previous findings from similar Simon tasks with children with ADHD [62,63]. Additionally, children tend to prioritise accuracy over speed, resulting in slower yet more accurate responses overall [60]. This pattern also highlights the importance of employing measures such as CAF analysis, which can reveal strong early impulsive tendencies not fully captured by overall error rates. Similarly to previous findings, ADHD children exhibited impaired inhibitory control, as revealed by delta plots [62]. Although in our study RT did not differ between ADHD and control groups, the CAF measure, which assesses RT, replicated previous findings by showing that children with ADHD made more errors during the fastest responses [63], indicating a stronger impulse to execute fast and inappropriate actions. Another limitation of the study is that in order to elicit robust temporal cueing effects in children, we induced temporal expectations with a flashing mobile phone stimulus rather than an arbitrary abstract cue as in previous studies [39]. The cue flashed twice in temporal cue condition whereas it flashed only once in the neutral condition, potentially causing differences in arousal in the two conditions. These differences in arousal could therefore explain performance differences in the control and ADHD groups [95]. However, in neurotypical children the effects of temporal versus neutral cues on mean RT and delta plots were very similar to those of long versus short FPs. Since long FPs are no more arousing than short FPs, and both long FPs and temporal cues carry temporally predictive information, our data suggest that the effects of temporal cues represent effects of temporal predictability rather than effects of arousal. More importantly, our key finding that children with ADHD made use of longer FPs to help suppress impulsive errors is based on group differences within the same neutral condition. Both short and long FPs were preceded by exactly the same cue (i.e., a single flash), and so cannot be due to differences induced by differentially arousing effects of single or double flashes.

In summary, this study reveals, for the first time, that ADHD children can voluntarily attend to and use symbolic temporal cues to speed their responses in a manner similar to neurotypical children. Furthermore, our results suggest that children with ADHD can leverage long preparatory intervals to better inhibit potentially erroneous impulses.
